# Lymphoma‐associated acquired von Willebrand syndrome responsive to splenectomy: A case report

**DOI:** 10.1002/jha2.486

**Published:** 2022-05-31

**Authors:** Fatima Khadadah, Natasha Rupani, Jordan Scott, Martina Trinkaus, Jerome Teitel, Michelle Sholzberg

**Affiliations:** ^1^ Division of Hematology Kuwait Cancer Control Centre Shuwaikh Kuwait; ^2^ Division of Hematology Department of Medicine University of Toronto Toronto Canada; ^3^ Division of Hematology/Oncology, Departments of Medicine and Laboratory Medicine & Pathobiology St. Michael's Hospital, University of Toronto Toronto Canada

**Keywords:** acquired von Willebrand syndrome, case report, hemostasis

## Abstract

A previously healthy 33‐year‐old female presented with a large hematoma over her right knee after kneeling. She was found to have pancytopenia and massive splenomegaly. Von Willebrand Factor (VWF) antigen level was 0.38 units/ml, ristocetin cofactor activity 0.13 units/ml, and VWF multimeric distribution was normal. Bone marrow examination revealed an indolent B‐cell lymphoma. Diagnosis was consistent with acquired von Willebrand syndrome as an autoimmune epiphenomenon of a lymphoma. Diagnostic and therapeutic splenectomy under hemostatic coverage was performed. VWF antigen levels and activities immediately normalized postoperatively and remained within the normal range several months later. Splenic pathology confirmed hairy cell leukemia with a BRAF mutation.

A previously healthy vegan 33‐year‐old female presented to St. Michael's Hospital, Toronto, Canada with an 8 × 8 cm hematoma over her right knee after kneeling during a CPR training session. She was found to have pancytopenia and massive splenomegaly measuring 40 cm. Over the previous year, she had heavy menstrual bleeding, atraumatic subcutaneous bleeding, and prolonged bleeding after dental extraction. There was no family history of excessive bleeding. Initial laboratory investigations revealed hemoglobin 77 g/L (reference 115–155), leukocytes 0.89 × 10^9^/L (3.5–10.5), neutrophils 0.2 × 10^9^/L (2.0–6.3), platelets 31 × 10^9^/L (130–380), ferritin < 10 µg/L (12–192), B12 106 pg/ml (133–630), prothrombin time 14 s (10–12), partial thromboplastin time 37 s (30–45), alanine aminotransferase 16 IU/L (10–49), alkaline phosphatase 22 IU/L (35–125), bilirubin 12 µmol/L (0–23), beta‐HCG negative.

The pancytopenia and splenomegaly raised concern of either an infectious etiology such as Epstein–Barr virus or a hematological malignancy such as splenic marginal zone lymphoma. The year‐long duration of symptoms suggested a chronic underlying process. The bleeding may have been due to thrombocytopenia; however, the extent of thrombocytopenia was discrepant with the marked degree of bleeding. The prothrombin time (PT) was mildly prolonged, and the activated partial thromboplastin time (aPTT) and fibrinogen were within normal limits. Her low B12 was thought to be due to her vegan diet. The 1 year bleeding history seemed to suggest an acquired bleeding disorder.

Infectious workup included negative blood cultures and negative viral serologies (hepatitis B, hepatitis C, Parvovirus, HIV, and EBV). Repeated coagulation studies revealed mildly prolonged PT (15 s) and aPTT time (45 s) along with several mild clotting factor deficiencies (fibrinogen 1.61 g/L, factor V 0.35 U/ml [ref 0.60–1.50 U/ml], factor VIII 0.31 U/ml, factor IX 0.52U/ml [ref 0.60–1.50 U/ml], factor XI 0.44U/ml [ref 0.50–1.50U/ml]). Clotting factors II, VII, X, and XII were within normal limits. Due to the mucocutaneous nature of her bleeding and low factor VIII, von Willebrand factor (VWF)‐related testing was performed, which showed a VWF antigen level of 0.38 units/ml (reference interval 0.50–1.50 IU/ml), ristocetin cofactor activity (RCof) of 0.13 units/ml (reference interval 0.50–1.50 IU/ml), and a normal VWF multimeric distribution. Blood group was O positive. IgG anti‐VWF antibodies (measured by a research ELISA) were weakly positive.

Considering the negative previous history of bleeding (including multiple dental extractions with no excess bleeding, and only recent onset of menorrhagia) and negative family history, the diagnosis of acquired von Willebrand syndrome (AVWS) was made with a type 2 M‐like pattern (discordant VWF activity to antigen ratio with normal VWF multimers). The most likely underlying cause was thought to be a hematological malignancy such as a lymphoma, which can cause AVWS through multiple immune and nonimmune mechanisms; myelofibrosis leading to splenomegaly seemed less likely given her age and the findings on complete blood count. The mild clotting factor deficiencies were thought to be related to the presence of massive splenomegaly resulting in mass compression of the liver and leading to hepatic synthetic dysfunction.

Bone marrow aspirate and biopsy revealed an indolent B‐cell lymphoma: hairy cell leukemia versus splenic marginal zone lymphoma. Massive splenomegaly without lymphadenopathy elsewhere was seen on computed tomography (Figure 1).

**FIGURE 1:**
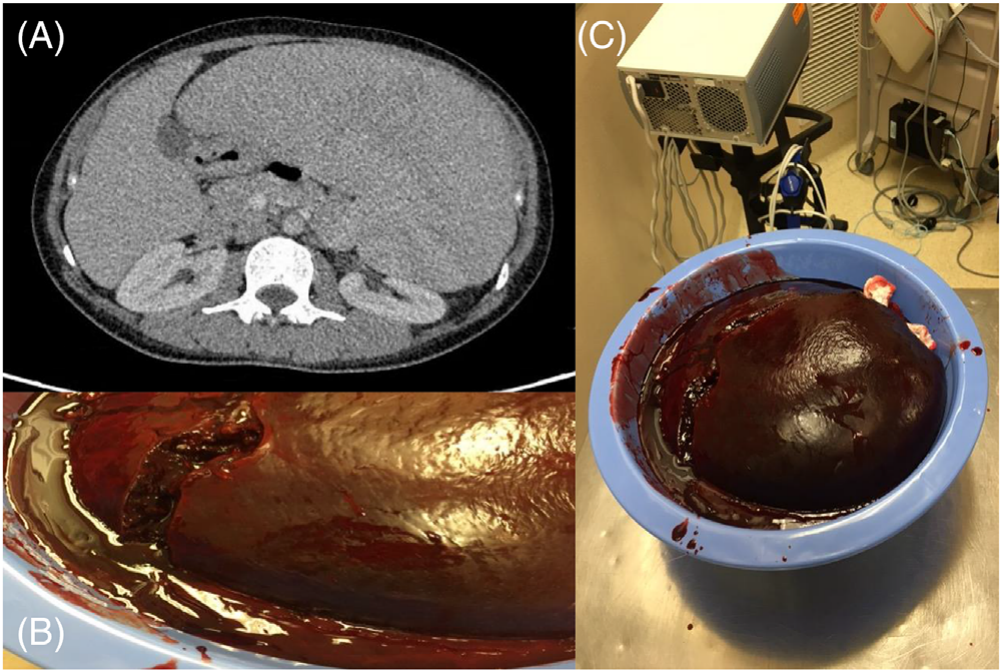
(A) CT abdomen‐image of massive splenomegaly. (B), (C) Massive spleen following resection

Given the size of the spleen, her pancytopenia, and symptoms, it was decided that she was likely to benefit clinically from splenectomy, which would also allow for a definitive diagnosis. Parenteral iron and vitamin B12 were provided for preoperative blood optimization due to heavy menstrual bleeding and her vegan diet. Tranexamic acid was provided to manage heavy menstrual bleeding. To help further manage bleeding and in preparation for upcoming splenectomy with consideration of AVWS and the weak anti‐VWF IgG suggestive of a possible immune‐mediated mechanism and moderate possibly immune thrombocytopenia (ITP), a 4‐day trial of pulse dexamethasone and two doses of intravenous immunoglobulin were given. No improvement was seen in the patient's VWF parameters or platelet count. Pharmacokinetic analysis after infusion of plasma derived VWF:FVIII concentrate showed that unusually large doses were required to maintain FVIII activity at or above 1.00 IU/ml, with a markedly shortened half‐life of 2 h (Figure 2).

**FIGURE 2:**
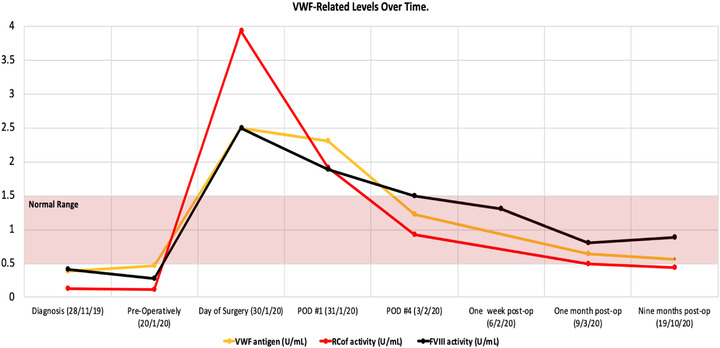
VWF‐related levels over time. VWF‐related testing at diagnosis, preoperatively and postoperatively following open splenectomy. POD: postoperative day; VWF: von Willebrand factor; RCOF: ristocetin cofactor activity; FVIII: factor eight activity. Normal range for variables shaded in red

The lack of improvement with dexamethasone and intravenous immunoglobulin were evidence against immune‐mediated AVWS or ITP. Improvement of VWF parameters with VWF:FVIII replacement was more consistent with a nonimmune mediated AVWS such as adsorption of VWF—in particular high molecular weight multimers—from plasma onto malignant cells [1]. However, irrespective of the mechanism, the most important step in AVWS management is treatment of the underlying disorder.

Laparoscopic splenectomy was performed under coverage with VWF:FVIII replacement. Her surgery had to be converted to open splenectomy due to the size of the spleen (Figure 1). Intraoperatively, she required massive doses of VWF:FVIII concentrate at 20 IU/kg/h (to maintain VWF RCof and FVIII levels of > 1.00 IU/ml), a single platelet pool transfusion, 3 g of fibrinogen concentrate (to achieve fibrinogen levels >2.0 g/L given preop fibrinogen of 1.58, intraop 1.65—1 day postop fibrinogen level was 2.61 without further supplementation) and tranexamic acid. With this hemostatic strategy, there were no perioperative bleeding complications.

Postoperatively, she did not require any additional VWF:FVIII concentrate, as there was abrupt and immediate complete normalization of her VWF parameters (Figure 2). Splenic pathology confirmed the diagnosis of hairy cell leukemia (positive BRAF mutation). There was near normalization of her mild clotting factor deficiencies on the first day postoperatively, without any plasma provided preoperatively, and complete normalization was observed by 6 weeks.

Her prognosis is expected to be excellent with a median 10‐year overall survival of greater than 90% [2]. The AVWS and other coagulopathies completely resolved with management of the underlying lymphoma.

## 1 COMMENTARY

While Von Willebrand disease (VWD) is the most common *inherited* bleeding disorder [3], AVWS is rare, more common in older age, and typically occurs in the absence of a previous personal or family history of bleeding [4]. VWF‐related testing can demonstrate quantitative and/or qualitative deficiency of VWF with patterns that simulate different subtypes of congenital VWD. The pathophysiology of AVWS can be divided into immune (via neutralizing or nonneutralizing antibodies) and nonimmune mechanisms (such as adsorption, increased cleavage, or decreased production) and is associated with a variety of lympho‐ and myeloproliferative disorders, solid tumors, autoimmune disorders, hypothyroidism, and cardiovascular conditions [5]. The bleeding phenotype is typically mucocutaneous and can lead to life‐threatening bleeding.

Investigation for AVWS is typically initiated in patients who present with new onset mucocutaneous bleeding and an AVWS‐associated condition. It is recommended that patients with a history suggestive of AVWS be referred to hematology for testing and management [6] at a specialized coagulation laboratory where the necessary resources are available, and where preanalytical variables that can lead to false positive or negative test results can be controlled [7].

With roughly 700 cases reported worldwide (case reports [8, 9], registry studies [4], other observational studies [10]), treatment is typically focused on the underlying condition and immediate correction of the hemostatic defect in the face of acute bleeding or need for urgent surgery. Such strategies are supported by small observational studies and anecdotal experience. In most cases, abnormalities in VWF levels improve or resolve when the underlying condition is treated [6]. Adjunctive therapies to correct hematinic deficiencies such as iron are imperative, and in this case B12, as this will help reduce transfusion requirements during active bleeding.

In the event of acute bleeding or surgery, hemostatic treatment options include (1) VWF factor replacement using VWF‐containing concentrates or desmopressin (which induces secretion of VWF from the Weibel–Palade bodies in endothelial cells); (2) in immune AVWS, immune modulation using intravenous immune globulin infusions, steroids or plasmapheresis; (3) bypassing therapy with recombinant activated factor VII; and (4) antifibrinolytic therapy. Reported hemostatic success with these approaches is variable and often a combination of modalities is required, depending on the underlying etiology.

Here we described a patient with severe AVWS associated with hairy cell leukemia, immediately and dramatically corrected by surgical removal of the patient's massive spleen. We postulate that the dramatically shortened half‐life of VWF in this patient may have been caused by VWF antibody‐mediated inhibition and/or VWF adsorption onto platelets sequestered in the spleen or onto the massive number of tumor cells. ELISA‐based testing for VWF‐antibodies both pre‐ and postoperatively was only weakly positive in a very sensitive research assay. Given this equivocal result and the rapid normalization of VWF levels postoperatively, we consider it more likely that our patient's AVWS was mediated by one of the adsorptive mechanisms described above. Surgical hemostasis required a combination of large doses of plasma derived VWF:FVIII concentrate and antifibrinolytic therapy. On‐site specialized coagulation laboratory testing with rapid turnaround time and access to specialized blood products were essential for diagnosis and monitoring, and for treatment to prevent life‐threatening bleeding.

## COMPETING INTERESTS

The authors have no competing interests. The authors have no funding to report for this submission.

## ETHICS STATEMENT

Local research ethics board approval and informed patient consent was obtained.
